# Underuse of medication for circulatory disorders among unmarried women and men in Norway?

**DOI:** 10.1186/2050-6511-15-65

**Published:** 2014-11-24

**Authors:** Øystein Kravdal, Emily Grundy

**Affiliations:** Norwegian Institute of Public Health, Oslo, Norway; Department of Economics, University of Oslo, Oslo, Norway; Department of Social Policy, London School of Economics and Political Science, London, UK; Department of Economics, P.O. Box 1095, Blindern, 0317 Oslo, Norway

**Keywords:** Norway, Circulatory disorders, Marital status, Medication, Underuse, Register data

## Abstract

**Background:**

It is well established that unmarried people have higher mortality from circulatory diseases and higher all-cause mortality than the married, and these marital status differences seem to be increasing. However, much remains to be known about the underlying mechanisms. Our objective was to examine marital status differences in the purchase of medication for circulatory diseases, and risk factors for them, which may indicate underuse of such medication by some marital status groups.

**Methods:**

Using data from registers covering the entire Norwegian population, we analysed marital status differences in the purchase of medicine for eight circulatory disorders by people aged 50-79 in 2004-2008. These differences were compared with those in circulatory disease mortality during 2004-2007, considered as indicating probable differences in disease burden.

**Results:**

The unmarried had 1.4-2.8 times higher mortality from the four types of circulatory diseases considered. However, the never-married in particular purchased less medicine for these diseases, or precursor risk factors of these diseases, primarily because of a low chance of making a first purchase. The picture was more mixed for the divorced and widowed. Both groups purchased less of some of these medicines than the married, but, especially in the case of the widowed, relatively more of other types of medicine. In contrast to the never-married, divorced and widowed people were as least as likely as the married to make a first purchase, but adherence rates thereafter, indicated by continuing purchases, were lower.

**Conclusion:**

The most plausible interpretation of the findings is that compared with married people, especially the never-married more often have circulatory disorders that are undiagnosed or for which they for other reasons underuse medication. Inadequate use of these potentially very efficient medicines in such a large population group is a serious public health challenge which needs further investigation. It is possible that marital status differences in use of medicines for circulatory disorders combined with an increasing importance of these medicines have contributed to the widening marital status gap in mortality observed in several countries. This also requires further investigation.

## Background

Numerous studies from many different countries have shown that married people have lower all-cause mortality than the unmarried and lower mortality from several specific causes and cause groups, including circulatory diseases [1–3]. Health-related and other types of selection to and from marriage are known to play a role in this differentiation [4], but marriage is also considered to have health protective effects. These include economic benefits [5], social support, social control by a spouse [6, 7] and, partly because of this social control, a lower propensity for risky behaviours [8–10]. These factors may affect health partly through differences in health-related behaviours more generally, including participation in screening programmes, medical consultation rates and adherence to medication, all of which would potentially lead to differences in medication use.

Our aim in this paper is to see whether there are differences by marital status in the purchase (encashment) of prescribed drugs for eight types of circulatory disorders. We also consider incident and continuing purchase of these medicines. These marital status differences in medication purchase (assumed to be indicative of medication use) are compared with marital status differences in mortality from four specific circulatory causes, used as an indicator of probable differences in disease burden. We use data from registers that cover the entire Norwegian population, and in particular the Norwegian Prescription Database, which includes all purchases of prescription medicine since 2004. The analysis covers the years 2004-2008.

Circulatory diseases are the most common group of causes of death in high income countries, so such an analysis of marital status differentials in the use of medicines for these diseases and their risk factors is potentially very important from a public health perspective. This is particularly the case in the context of improvements in the efficacy of therapeutic treatments for circulatory diseases. Advances in drug-based and surgical treatments have been found to matter greatly at the population level [11, 12] and perhaps explain ¼ to ½ of the reduction in the mortality from circulatory diseases over a couple of decades from about 1980 [13–15], and an American study showed that many years of life are lost because a large proportion of the population receive inadequate treatment for coronary heart diseases [16]. Analyses of differentials in medication use by marital status may thus also shed light on recent increases in marital status differentials in circulatory disease mortality, which have contributed strongly to the increases in the corresponding all-cause mortality differentials observed in a number of countries [17–19]. In Norway, especially the never-married have experienced a rising excess mortality compared to the married [17].

Previous research on marital status differentials in medication use is sparse and inconclusive. Delayed diagnosis or treatment for cardiovascular problems among unmarried groups has been reported in some studies [20, 21], which accords with studies showing that unmarried people are often diagnosed later with cancer [22, 23] and have lower participation rates in screening for various diseases [24]. Differences in adherence to treatments for various diseases by marital status and availability of social support have also been reported. For example, some studies have shown that unmarried people are less likely to follow doctors’ advice about medication after diagnosed coronary diseases [25–28] or hypertension [29], and adverse changes in health care utilisation among recently widowed chronically ill men have been reported as well [30]. However, other recent studies of adherence to treatment for circulatory diseases or conditions (risk factors) have not found marital status differences [31, 32]. Moreover, the evidence – in either direction - is generally relatively weak because of small sample sizes [28, 29, 31] and possible bias arising from use of self-reported measures of medication adherence [25, 29, 31], rather than measures from prescription registers [32] or electronic monitoring [28].

## Methods

### Data

The study is based on analysis of two data files, constructed (in 2009-2012) from various Norwegian population registers. Both include, for everyone who has lived in Norway since 1960, year of death, immigration and emigration (if any) and marital status and educational level at the beginning of each year (since 1980, though only the information for the years after 2004 was used in the analysis). These variables were taken from the Central Population Register and the Educational Database (operated by Statistics Norway). Additionally, one of the files includes information from the Cause-of-death Register (up to 2007), while the other includes information from the Norwegian Prescription Database (NorPD) for 2004-2008. NorPD covers all purchases of prescription medicine (defined by Anatomical Therapeutic Chemical (ATC) classification) since 2004 by Norwegian residents, except individuals living in institutions [33]. Because of the latter exclusion, we restricted our analysis to persons younger than 80 during the period under study. The lowest age considered is 50. While circulatory diseases occur among younger individuals, and are treated medically, there are few deaths from this cause group at these lower ages.

Separate analyses were undertaken for women and men, as several studies have suggested that some of the health benefits of partnership may be sex-specific, especially in older age groups [34, 35]. In all models, we controlled for level of education, which is an important determinant of marriage and divorce [36, 37] as well as health [38] and health care use [39]. Age was, of course, also controlled for.

The use of register data for this research purpose has been approved by the Regional Committees for Medical and Health Research Ethics and the Norwegian Data Protection Authority.

### Mortality analysis

Discrete time hazard models for all-cause and cause-specific mortality were estimated for the period 2004-2007, following standard procedures [40]. A series of one-year observations was constructed, starting in January 2004, for all those then aged 49-78 and living in the country. The last observation was the year of death, the year the person turned 79, the year of emigration, or 2007, whichever came first. Those who became 50 or immigrated during the observation period were added from January of the year of their 50^th^ birthday or the year after immigration, respectively. Observations were excluded if the person lived temporarily abroad at the beginning of the year. Logistic models were estimated from all remaining observations. In total, there were 35,174 deaths within 2,602,246 person-years of observation among men and 23,970 deaths within 2,693,670 person-years of observation among women. Table 1 shows the distribution of these by marital status.

**Table 1:** **Number of deaths and person-years of exposure in the mortality analysis of women and men aged 50-79 in 2004-2007, by marital status**

	Never-married	Married	Widowed	Divorced/separated
**Men**				
Number of deaths from all causes	5325	19995	3321	6533
Number of deaths from all circulatory diseases	1679	6031	1145	2025
Person-years of exposure	277508	1818415	99705	406618
**Women**				
Number of deaths from all causes	2027	10515	7490	3938
Number of deaths from all circulatory diseases	509	2248	2262	829
Person-years of exposure	175001	1670393	400626	447650

The specific causes of death considered are ischemic heart diseases (ICD-10 codes I00-I25), other heart diseases (I26-I52), cerebrovascular diseases (I60-I69) and all other circulatory diseases (other I00-I99).

### Analyses of medicine purchases: prevalence, incidence and discontinuation

In the analysis of drug purchases we considered eight groups of drugs, defined by Kuo et al. [41] as indicating treatment for specific disorders. These disorders were (ATC codes shown in notes to Table 2): i) coronary and peripheral vascular diseases treated with platelet aggregation inhibitors, ii) coronary and peripheral vascular diseases treated with anticoagulants, iii) hypertension without any diagnosed heart disease, iv) hyperlipedemia, v) arrhythmic cardiac diseases, vi) angina, vii) congestive heart failure (usually in combination with hypertension), and viii) other ischemic heart diseases (usually in combination with hypertension). The analysis included three steps. First, logistic models for the chance of purchasing certain drugs at least once during 2004-2008 (i.e. prevalence of medication purchase and, presumably, use) were estimated, conditional on living in the country at the beginning and end of that period. There were 579,218 observations for men and 620,911 for women.

**Table 2:** **Effects of marital status on all-cause and cause-specific mortality (ICD-10 codes in parentheses) 2004-2007** ^**a**^ **and the chance of purchasing various types of medicines for circulatory disorders 2004-2008 among women and men aged 50-79** ^**b**^

		Effects of marital status (with 95% confidence intervals)			Number of deaths or persons purchasing the medicines
		Never-married	Widowed	Divorced/separated	
**Men**					
Mortality from	All causes	2.05*** (1.98-2.11)	1.56*** (1.50-1.62)	1.96*** (1.91-2.02)	35174
	All circulatory diseases (I00-I99)	2.11*** (1.99-2.23)	1.65*** (1.54-1.75)	2.08*** (1.97-2.18)	10880
	Ischemic heart diseases (I20-I25)	1.94*** (1.80-2.10)	1.67*** (1.52-1.82)	1.93*** (1.80-2.07)	5737
	Other heart diseases (I26-I52)	2.71*** (2.39-3.08)	1.63*** (1.52-1.82)	2.52*** (2.24-2.85)	1809
	Cerebrovascular diseases (I60-I69)	1.99*** (1.75-2.26)	1.51*** (1.31-1.74)	2.07*** (1.84-2.33)	2066
	Other circulatory diseases	2.13*** (1.81-2.50)	1.75*** (1.46-2.10)	2.11*** (1.82-2.10)	1268
Purchase of medication for	Coronary and peripheral vascular diseases, antiplatelet^c^	0.70*** (0.68-0.73)	1.00 (0.95-1.05)	1.02 (0.99-1.05)	42780
	Coronary and peripheral vascular diseases, anticoagulant^d^	0.88*** (0.85-0.91)	1.04* (1.00-1.08)	1.03** (1.00-1.06)	59898
	Hypertension^e^	0.98 (0.95-1.01)	1.10*** (1.06-1.15)	0.97*** (0.94-0.99)	56019
	Hyperlipedemia^f^	0.69*** (0.68-0.71)	0.95*** (0.93-0.98)	0.89*** (0.87-0.90)	185023
	Anti-arrhythmic cardiac diseases^g^	1.02 (0.97-1.08)	1.09*** (1.03-1.16)	1.09*** (1.04-1.14)	18204
	Ischemic heart diseases/angina^h^	0.75*** (0.73-0.78)	0.98 (0.94-1.02)	0.99 (0.97-1.02)	65918
	Congestive heart failure/hypertension^i^	0.92*** (0.90-0.94)	1.10*** (1.07-1.32)	0.96*** (0.95-0.98)	195657
	Other ischemic heart diseases/hypertension^j^	0.88*** (0.86-0.90)	1.08*** (1.05-1.11)	0.96*** (0.94-0.97)	198133
**Women**					
Mortality from	All causes	2.05*** (1.95-2.15)	1.40*** (1.35-1.44)	1.70*** (1.64-1.77)	23970
	All circulatory diseases (I00-I99)	2.37*** (2.15-2.61)	1.53*** (1.44-1.62)	1.83*** (1.69-1.98)	5848
	Ischemic heart diseases (I20-I25)	2.38*** (2.05-2.77)	1.44*** (1.31-1.59)	1.80*** (1.59-2.04)	2323
	Other heart diseases (I26-I52)	2.84*** (2.30-3.50)	1.66*** (1.44-1.90)	2.14*** (1.79-2.56)	1163
	Cerebrovascular diseases (I60-I69)	2.18*** (1.82-2.61)	1.51*** (1.35-1.69)	1.63*** (1.40-1.91)	1678
	Other circulatory diseases	1.94*** (1.43-2.64)	1.63*** (1.36-1.95)	1.91*** (1.36-1.95)	684
Purchase of medication for	Coronary and peripheral vascular diseases, antiplatelet ^c^	0.84*** (0.78-0.90)	1.12*** (1.08-1.16)	1.24*** (1.19-1.29)	22058
	Coronary and peripheral vascular diseases, anticoagulant^d^	0.92*** (0.88-0.96)	1.07*** (1.04-1.10)	1.08*** (1.05-1.11)	51264
	Hypertension^e^	0.96* (0.93-1.00)	1.03*** (1.01-1.06)	0.94*** (0.92-0.97)	58882
	Hyperlipedemia^f^	0.78*** (0.77-0.81)	0.95*** (0.94-0.97)	0.92*** (0.91-0.94)	175477
	Anti-arrhythmic cardiac diseases^g^	1.00 (0.92-1.09)	1.08*** (1.03-1.13)	1.07** (1.01-1.14)	11746
	Ischemic heart diseases/angina^h^	0.85*** (0.82-0.89)	1.11*** (1.08-1.13)	1.20*** (1.16-1.23)	52641
	Congestive heart failure/hypertension^i^	0.95*** (0.93-0.97)	1.07*** (1.06-1.09)	0.99 (0.97-1.01)	203305
	Other ischemic heart diseases/hypertension^j^	0.87*** (0.85-0.89)	1.03*** (1.01-1.04)	0.94*** (0.92-0.95)	187899

^a^Discrete-time hazard models are estimated. The models also include age (in five-year groups), year (in one-year groups), and educational level (in five categories). *p < 0.10; **p < 0.05; ***p < 0.01.

^b^Logistic models for the chance of purchasing the medicines are estimated. The models also include age (in five-year groups) and educational level (in five categories). Medicines are grouped as in Kuo et al. [41]. See details in notes c-j.

^c^ATC codes B01AC, C04AD03 (except B01AC06, B01AC08, B01AC09, B01AC11, B01AC15, B01AC19, B01AC21).

^d^ATC codes B01AA, B01AB, B01AD, B01AX.

^e^ATC codes C02AA02, C02AB02, C02AC, C02BA, C02BB, C02CA, C02CC, C02DA, C02DB, C02DD, C02DG, C03AA, C03AB, C03AX, C03DA, C03DB, C02L.

^f^ATC codes C10AA, C10AB, C10AC, C10AD, C10AX, C10BA, C10BX.

^g^ATC codes C01AA, C01BA, C01BB, C01BC, C01BD, C01BG, C01EB10.

^h^ATC codes C01DA, C01DX16.

^i^ATC codes C01CA07, C01CE01, C01CE02, C01EB09, C03CA, C03CB, C03CC, C09AA, C09BA, C09BB, C09CA, C09DA, C09DB.

^j^ATC codes C07AA, C07AB, C07AG, C07BA, C07BB, C07BG, C07CA, C07CB, C07CG, C07DA, C07DB, C07EA, C07EB, C07FA, C07FB, C08CA, C08CX, C08DA, C08DB, C08EA, C08EX, C08GA.

In the second step, discrete-time hazard models for the chance of starting to purchase the specific type of drug (i.e. incidence) were estimated for those who did not use it in 2004. More specifically, one-year observations from 2005 were created conditioned on the individual being of age 50-79 that year, being alive both at the start and the end of the year and not yet having started to purchase the medicine by the beginning of the year. The outcome variable was whether the drug was purchased at least once during the year.

Similarly, in the third step, discrete-time hazard models for discontinuing the purchase of the medicine were estimated. One-year observations from 2005 were created conditioned on the individual being of age 50-79 that year, being alive both at the start and the end of the year, having purchased the medicine at least once during the preceding year, but having had no earlier discontinuation of the purchase of that medicine (i.e. the individual has either purchased the medicine in every year from 2004 to the preceding year, or started in 2005 or later and kept purchasing until at least the previous year). The outcome variable was whether the drug was purchased at least once during the year. For simplicity, the signs of the effects are reversed in the tables and can thus be interpreted as effects on continuation rather than discontinuation.

## Results

### Marital status differences in mortality from circulatory diseases

Mortality from circulatory disease was more than twice as high among the never-married as among the married for both men and women. For all circulatory diseases combined the odds ratio for men was 2.11 (95% CI 1.99-2.23) and that for women 2.37 (2.15-2.61). Odds ratios for men and women varied between 1.94 and 2.84 across the four main sub-categories of circulatory diseases considered (Table 2). For men the estimates were 1.93-2.52 for the divorced and 1.51-1.75 for the widowed. Similar results were seen for women, with odds ratios of 1.63-2.14 for the divorced, and 1.44-1.66 for the widowed.

### Associations between marital status and purchase of medication 2004-2008

Never-married men and women were generally less likely than the married to purchase medicine for circulatory disorders (Table 2 and signs shown in Panel A of Table 3). The only exceptions were that there was no significant association between being never-married and purchasing medicine for hypertension (alone) or arrhythmic diseases. For both sexes, the sharpest negative effects were seen with respect to drugs for hyperlipidemia.

**Table 3:** **Sign and significance (+ and - meaning p <0.10; ++ and - - meaning p <0.05; +++ and - - - meaning p <0.01) of effects of marital status on the chance of purchasing medicine in 2004-2008, the chance of starting to purchase medicine 2005-2008, and the chance of continuing with the medicine purchases 2005-2008**

		Men Never-married	Widowed	Divorced/separated	Women Never-married	Widowed	Divorced/separated
**Panel A: Purchase of medicine in 2004-2008 (signs as in estimates in Table ** 2 **)**							
Coronary and peripheral vascular diseases, antiplatelet		- - -			- - -	+ + +	+ + +
Coronary and peripheral vascular diseases, anticoagulant		- - -	+	+ +	- - -	+ + +	+ + +
Hypertension			+ + +	- - -	-	+ + +	- - -
Hyperlipedemia		- - -	- - -	- - -	- - -	- - -	- - -
Anti-arrhythmic cardiac diseases			+ +	+ +		+ + +	+ +
IHD/Angina		- - -			- - -	+ + +	+ + +
Congestive heart failure/hypertension		- - -	+ + +	- - -	- - -	+ + +	
Other IHD/hypertension		- - -	+ + +	- - -	- - -	+ + +	- - -
**Panel B: Starting and continuing purchase of medicine 2005-2008** ^**a**^							
Coronary and peripheral vascular diseases, antiplatelet	Start	- - -		+ + +	- - -	+ + +	+ + +
	Continuation	+ +			+ + +		+ + +
Coronary and peripheral vascular diseases, anticoagulant	Start	- - -		+ + +		+ + +	+ + +
	Continuation			- - -	+		
Hypertension	Start		+ + +		-	+ +	-
	Continuation	+ + +		- - -	+ +		- - -
Hyperlipedemia	Start	- - -			- - -	- -	-
	Continuation	- - -	- - -	- - -		- - -	- - -
Anti-arrhythmic cardiac diseases	Start		+ + +	+ + +		+ + +	+ + +
	Continuation	+ + +		- -			
IHD/Angina	Start	- - -			- - -	+ + +	+ + +
	Continuation	+ + +			+ + +	+	
Congestive heart failure/hypertension	Start	- - -	+ + +	+ + +		+ + +	+ +
	Continuation	- - -	- - -	- - -	- - -	- - -	- - -
Other IHD/hypertension	Start	- - -	+ + +	+ + +	- - -	+ + +	+ +
	Continuation		- - -	- - -			- - -

^a^Discrete-time hazard models are estimated. The models also include age (in five-year groups), year (in one-year groups), and educational level (in five categories).

Among divorced men and women, the purchase of four types of medicine was lower than in the married reference group. These were medication for hypertension (alone), hyperlipidemia, heart failure (among men), and ischemic heart disease. On the other hand, three types of medicine were purchased to a larger extent by the divorced than the married, for one or both sexes: medicine for coronary and peripheral vascular diseases (antiplatelet as well as anticoagulant treatment), arrhythmic diseases and angina. However, with two exceptions (1.20 and 1.24), the positive estimates were not above 1.10.

The widowed were, on the whole, more likely than the married to purchase medicine for circulatory disorders. There was only one negative relationship: the use of medicine for hyperlipidemia. Relationships with respect to several other disorders were positive: hypertension, arrhythmic diseases, heart failure and ischemic heart disease for both sexes and coronary and peripheral vascular diseases and angina for women. Again, the positive relationships were not large; all effects were smaller than 1.12.

### Associations between marital status and starting and continuing drug purchase

The lower chance of purchasing medicine among the never-married reflects a lower chance of making a first purchase (Table 3, Panel B). Once initiated, the chance of continuing to purchase the medication was significantly higher than (at a 5% level) or as high among the never-married as the married for several disorders, exceptions being heart failure and, for men, hyperlipedemia.

For the divorced, who generally purchased about as much medicine for circulatory disorders as the married, there is an almost opposite pattern. Their chance of starting to purchase medicine was quite *high*. This was seen for several types of medicine for both men and women, and no effect runs in the other direction. On the other hand, they had a relatively *low* probability of *continuing* to purchase several types of the medicines considered, and there was only one example of the opposite.

The pattern for the widowed was similar to that for the divorced: there were several positive associations with the chance of starting to purchase medicine and one negative (as opposed to none for the divorced), and there was no positive association with the chance of continuing the purchases (as opposed to one for the divorced) and several negative ones. However, there were fewer of the latter associations (five in total, for three different conditions, as opposed to 10 in total for the divorced), so on the whole, the widowed appear to be less different from the married than are the divorced.

## Discussion

These results show, consistent with other studies, large marital status variations in mortality from circulatory diseases with the highest risks, relative to married people, for the never-married followed by the divorced and then the widowed. Overall, in the age group considered here (50-79), the unmarried had rates of mortality from circulatory diseases 1.4-2.8 times higher than those of the married. However, never-married people in particular purchased less of the medicines that are typically used after such a circulatory disease has been diagnosed or risk factors for it identified. This was primarily because of a significantly lower chance of first purchase of most of these medicines The picture is more mixed for the divorced and widowed, who purchased less of some of these medicines but more of others, though the difference in the latter direction, which was seen especially among the widowed, was quite small (but statistically significant). In contrast to the never-married, the chances of starting to purchase these medicines were as least as high among the divorced and widowed as among married people, but adherence rates thereafter, indicated by continuing medication purchases, were lower (especially among the divorced).

We assume that purchases of medication are good measures of actual use of medication, and therefore refer to use in the remaining discussion. To the extent that there is a difference between purchase and use, it would seem likely, if anything, that the unmarried might be less inclined than the married to use the purchased medication, because of the lack of social support and control by a spouse that is further discussed below. If so, marital status differences in actual use may be more pronounced than suggested by our analysis of purchases.

### Interpretations of the observed patterns: underuse of medication?

To draw conclusions about underuse of medicine one should ideally compare actual use with need or recommended use. Unfortunately, we lack information on the actual prevalence and severity of the various circulatory disorders (and associated risk factors), which would be good indications of the need, and must instead compare differences in medication purchase, reflecting medication use, with those in mortality. As mentioned, one of our key findings is that the never-married use less medicine than the married for all disorders considered except a few. In theory, it is possible that they also *need* less of these medicines, despite their much higher mortality from all circulatory disease groups (and thus presumably also from the disorders under consideration). Let us assume that they to a larger extent than the married suffer from the *most severe* types of each of these disorders, but have a lower prevalence of the *less* severe types for which medication is nevertheless recommended. Then, even if they take medicine in accordance with recommendations or needs (i.e. no underuse), they could end up using less medicine than the married and have higher mortality. In other words, low usage coupled with high mortality does not *necessarily* mean underuse.

However, this situation seems improbable especially as there are common risk factors for many less and more serious conditions and evidence from numerous studies points to a higher, rather than lower, prevalence of circulatory diseases and associated risk factors among the unmarried than the married. More specifically, it has been shown that the unmarried have higher cholesterol levels than the married [42], higher prevalence of diabetes [43, 44], and higher blood pressure [42, 45–47], although other studies do not point so clearly in this direction [48–52]. A modest excess prevalence of stroke among the unmarried has also been reported [52, 53], as well as an excess prevalence of heart failure [54]. Consistent with this, other studies have reported that lifestyle factors associated with higher circulatory disease risks are less common among married people. In particular, the unmarried are more likely than the married to be physically inactive [42, 49, 50] (but see [55] for an opposite result), to be obese [49, 50, 56], (but see [55] for an opposite result) to have a high intake of sodium [46], to eat more fast food [55] and to smoke [42, 43, 49].

Theoretically, the never-married could also have a lower prevalence of both the more and the less severe types of any disorder - thus taking less medicine even without any underuse - and still have a strong mortality disadvantage because of less adequate use of *other* types of treatment, such as angioplasty or bypass surgery. However, a study by Bearden et al. [57] that addressed the latter issue did not show any relationship between marital status and these kinds of surgical interventions. To conclude, we think the most plausible interpretation of our findings is that the unmarried, and especially the never-married, suffer from circulatory disorders to a larger extent than the married and therefore have a greater need for medication, but that they are on the whole less likely than the married to take the medication they need, which adds further to their mortality disadvantage.

If the unmarried are particularly inclined to use less medicine than recommended or needed for their circulatory disorders, the implications for mortality depend on whether this underuse is most pronounced with respect to the less severe or the more severe types of the disorder. The use of statins (for hyperlipidemia) may serve as a particularly relevant example. This drug is used both as primary prevention (i.e. in the absence of actual circulatory diseases and even at rather low cholesterol levels if other risk factors are identified) and secondary prevention (i.e. when a circulatory disease is diagnosed and presumably treated). The efficacy of this primary prevention has been highly disputed, though a recent Cochrane review pointed towards a mortality reduction [58], which would justify continuation of the practice. We have found that the married use more of this medicine than all groups of unmarried, despite the evidence summarised above that suggests higher cholesterol levels for the unmarried and thus more need for this medicine. Hypothetically, however, it could be that use of statins among the unmarried for the purposes of secondary prevention is consistent with their needs – and so higher than use among the married- whereas use for the purposes of primary prevention is much lower. Such a pattern would have fewer implications for mortality risks than a lower than recommended use for secondary prevention.

Finally, it should be noted that, if the unmarried to a larger extent than the married take too little medicine compared to their needs, they may of course still use somewhat more medicine per person. For example, we have found that some groups of unmarried use more medicine for arrhythmic diseases than the married, and no group less, which may reflect that there is a particularly large difference between the married and the unmarried in the actual prevalence of that disorder. The fact that mortality from “cardiovascular diseases other than ischemic heart diseases” varies particularly much according to marital status lends some support to this idea. Similarly, the unmarried use more diuretics for heart failure (a subgroup of medicines for that disease and not shown in the tables), alone or in combination with other medicine, and this may indicate a relatively high prevalence of right-sided heart failure, which may be linked to chronic obstructive pulmonary disease and thus smoking – the latter being more common among the unmarried according to some studies as already noted [42, 43, 49].

### Factors underlying the differentials in use, and likely underuse, of medication

As mentioned above, studies of marital status differences in health and mortality consistently refer to economic benefits of marriage and the important role played by spouses in providing social support and exerting social control [5–7]. Such factors are thought to affect the disease or accident incidence - through lifestyle (including the use of explicit preventive strategies) - as well as the availability of care, use of medical treatment and other factors of importance for the further development of the diseases. The pathway of special relevance given our perspective is that a spouse’s encouragement and monitoring – but perhaps not the more favourable economic position of the married - may affect the chance of seeking a consultation, receiving a diagnosis (or identifying risk factors) and prescription, cashing the initial prescription, and seeking and cashing further prescriptions. The reason for the probably modest importance of economic resources is that costs of prescriptions are typically not very high and for those with chronic diseases, there are subsidies as well as a cap on total annual expenses in Norway.

The evidence on such causal pathways is not large, but some studies of cardiovascular diseases suggest that spouses may encourage attendance at screening, prompt consultation after onset of symptoms or otherwise increase the chance of an early diagnosis [20, 21]. Consistent with this, other studies have shown that the unmarried are often diagnosed later with cancer [22, 23] and have lower participation rates in screening for various diseases [24]. Some, but not all, earlier investigations have also shown a poorer treatment adherence among the unmarried – within rather small patient samples and often based on self-reporting of drug use [26–28, 31, 32, 59], see details in Introduction. One recent very large study found no differences between partnered and unpartnered women in reported use of common medications for IHD two years after first hospital admission for this condition [60], but it may be that differences in adherence are less marked for severe conditions which have resulted in acute admissions.

In the absence of a spouse, children may play a similar role in supporting consultation and treatment adherence, and as fewer of the never-married than the widowed or divorced have children, this might account for some of the differences we found between groups of the unmarried when it comes to the chance of using and starting to use the medicines for circulatory disorders. However there is little evidence from previous studies on this issue. Another possible reason for these differences between the never-married and formerly married is that the latter may persist with habits of health care seeking developed during their married life, though it is harder to understand why they are more inclined to *discontinue* the use of medicines.

### Limitations and strengths of the analysis

The study has some limitations, the most important being the lack of information on actual disorders as discussed above. Furthermore, formal marital status does not tell us the full story about actual living arrangements. Some of the unmarried live in cohabiting unions (currently 29% in the age group 45-66 and 10% in the age group 67-79, according to Statistics Norway [61]), and if cohabitants use medicine to a larger extent than the unmarried who live alone and do not have a partner, the difference between the latter and the married will be even larger than the estimated difference between the unmarried and the married that we report. Some authors have shown that the lower prevalence of circulatory diseases or their risk factors among the married is restricted to those having a good marriage, whereas those in a poor marriage are in a worse position than the single [51, 62, 63]. With register data, it is of course impossible to consider the quality of the marriage. We also lacked data on other possibly relevant confounders, mediators or moderators such as lifestyle factors, values, prior health status or indicators of socio-economic status other than education.

Additionally, NorPD does not include drug purchases for individuals in health care institutions. Since the unmarried are more likely to be institutionalized, the actual use of medicine in this group may not be quite as modest compared to the married as indicated by our estimates. However, this bias must be small, as the analysis only includes persons younger than 80, and at age 75-79 only 5% live in institutions [64].

An important strength of the study is availability of data that covers the whole population in which the measure of drug use was based on recorded purchase, assumed to be a good indicator of use, rather than self-report of use.

## Conclusion

Our results suggest that there is an underuse of medicine for circulatory disorders among the unmarried, and especially the never-married. The low usage of medication by the never-married particularly reflects low initial purchase of medication whereas for the divorced and widowed failure to continue with medication seemed more important. Inadequate use of these potentially very efficient medicines in such a large population group is, of course, a serious public health problem. Furthermore, underuse of medication for circulatory disorders among the unmarried in relatively recent years, combined with an increasing importance of such medicines, suggest a growing disadvantage for the unmarried when it comes to this type of medication (unless there was a much more pronounced underuse among these groups in earlier years, when the available medication was less efficient). This increasing disadvantage may have contributed to their falling behind with respect to mortality from circulatory diseases, and thus the widening marital status gap in all-cause mortality – which has been seen in a number of countries. In Norway, the excess mortality has increased particularly much for the group of unmarried also showing the clearest indications of underuse of medication for circulatory disorders, the never-married. Stated differently, the unmarried – and particularly the never-married- may not have benefited from recent life-saving improvements in medical treatment for circulatory disorders to the same extent as the married.

Further investigation of this issue is clearly warranted. A central element of such investigations should be to collect data that make it possible to better measure the potential underuse of medication. For that purpose, one would need information about the actual prevalence of the disorders as well as all types of treatment that are used. Obviously, it would also be an advantage if the data could shed light on the underlying mechanisms such as, for example, the role of spouses and children in prompting seeking of and adherence to treatment.
